# Intradermal methylene blue analgesic application in posthemorrhoidectomy pain management: a randomized controlled trial

**DOI:** 10.3389/fsurg.2024.1354328

**Published:** 2024-03-21

**Authors:** Ramin Azhough, Pooya Jalali, Mohammad Reza Dashti, Sahar Taher, Ali Aghajani

**Affiliations:** ^1^ Department of General Surgery, Faculty of Medicine, Tabriz University of Medical Sciences, Tabriz, Iran; ^2^ Basic and Molecular Epidemiology of Gastrointestinal Disorders Research Centre, Research Institute for Gastroenterology and Liver Diseases, Shahid Beheshti University of Medical Sciences, Tehran, Iran; ^3^ Faculty of Medicine, Tabriz University of Medical Sciences, Tabriz, Iran; ^4^ Faculty of Medicine, Islamic Azad University Tabriz Branch, Tabriz, Iran; ^5^ School of Medicine, Ahvaz Jundishapur University of Medical Sciences, Ahvaz, Iran

**Keywords:** methylene blue, hemorrhoidectomy, postoperative pain, numeric rating scale, intradermal injection

## Abstract

**Introduction:**

Unbearable post-hemorrhoidectomy pain is a well-documented challenge, significantly impacting patient well-being and satisfaction after surgery, often influencing patients to decline in undergoing this procedure. It is widely recognized that methylene blue has an effect of reducing inflammation and pain by reduces the production of nitric oxide and inhibiting the action potentials production in nerves. This study aims to explore the potential benefits of postoperative regional administration of methylene blue in providing extended relief from post-hemorrhoidectomy pain.

**Methods:**

This study included 97 patients aged 18–75 undergoing hemorrhoidectomy for stage III or IV hemorrhoids. A double-blind, randomized controlled trial compared postoperative intradermal injections of 1% methylene blue to 0.5% Marcaine as the control group. Two-week follow-up assessed pain. Statistical analysis, adherence to ethical standards, and registration were conducted.

**Result:**

No significant differences were found in baseline demographics, surgical parameters, or complications between the Methylene Blue and control groups. Intervention group remained lower in mean pain score until the 12th day. Methylene blue group reported significantly lower postoperative pain scores from days 1 to 7, with no significant differences afterward.

**Conclusion:**

This ongoing randomized controlled trial reveals the potential analgesic benefits of intradermal injection 1% methylene blue. It demonstrates comparable efficacy in reducing post-hemorrhoidectomy pain, with negligible side effects and complications.

## 1 Introduction

Unbearable post-hemorrhoidectomy pain is an expected event that results in patient discomfort and increased financial burdens by extended hospitalization. The causes of the pain following hemorrhoidectomy are diverse (1). Hemorrhoidectomy is typically recommended for individuals with grade III or IV hemorrhoids (2). Nevertheless, the prevalence of postoperative incision pain is high, serving as a significant factor leading patients to decline surgery (3). It is widely recognized that methylene blue reduces the production of nitric oxide (NO) by directly inhibiting the expression of endothelial nitric oxide synthase (eNOS). Additionally, it hinders the conversion of guanosine triphosphate (GTP) to cyclic guanosine monophosphate (cGMP) by suppressing the expression of soluble guanylate cyclase (sGC) in vascular smooth muscles. This dual mechanism ultimately results in vasoconstriction (4, 5). Nevertheless, in pathological conditions, there is an overexpression of NO, leading to its role as a pro-inflammatory mediator that contributes to inflammation (6, 7). importantly, through the direct downregulation of inducible nitric oxide synthase (iNOS), methylene blue effectively inhibits the inflammatory signaling mediated by iNOS (8). Furthermore, methylene blue might play a role in diminishing pain by impeding or causing damage to nerve connections in tissues, a process known as denervation. In fact, it has the potential to render affected nerve fibers or neurons incapable of perceiving pain (9). In the treatment of pruritus ani, two independent studies employed subcutaneous methylene blue. Even after a long-term period with no distinct nerve endings detected in the samples, the application of methylene blue was reported as successful, without major complications such as incontinence (1, 10). The first attempt to alleviate pain through intradiscal methylene blue injection in patients with chronic discogenic low back pain was conducted by Peng et al. The majority of patients exhibited promising outcomes, and this relief persisted for a minimum of 1 year (11). The pivotal role of voltage-gated sodium channels (VGSCs) and the generation of action potentials (AP) in pain transmission has been emphasized (12, 13). Intriguingly, ample evidence highlights methylene blue's ability to silence excitable cells or nerves by significantly reducing inward sodium currents (I_NA_) and inhibiting AP production (9, 14–17). In this regard, in a study by Sim, H. L. and K. Y. Tan, the combination of methylene blue and ropivacaine before dissection has exhibited a significant analgesic effect, particularly evident in the initial three days post-surgery. The probable mechanism of action involves the destruction of dermal nerve endings by methylene blue (18). In this present study, we aim to investigate the potential benefits of postoperative administration of regional methylene blue in providing extended relief from postoperative pain following hemorrhoidectomy. To achieve a comprehensive understanding of its efficacy, we have designed a comparative analysis; the pain-relieving effects of intradermal methylene blue against Marcaine (bupivacaine) injection.

## 2 Materials and methods

### 2.1 Protocol and registration

**Figure 1:**
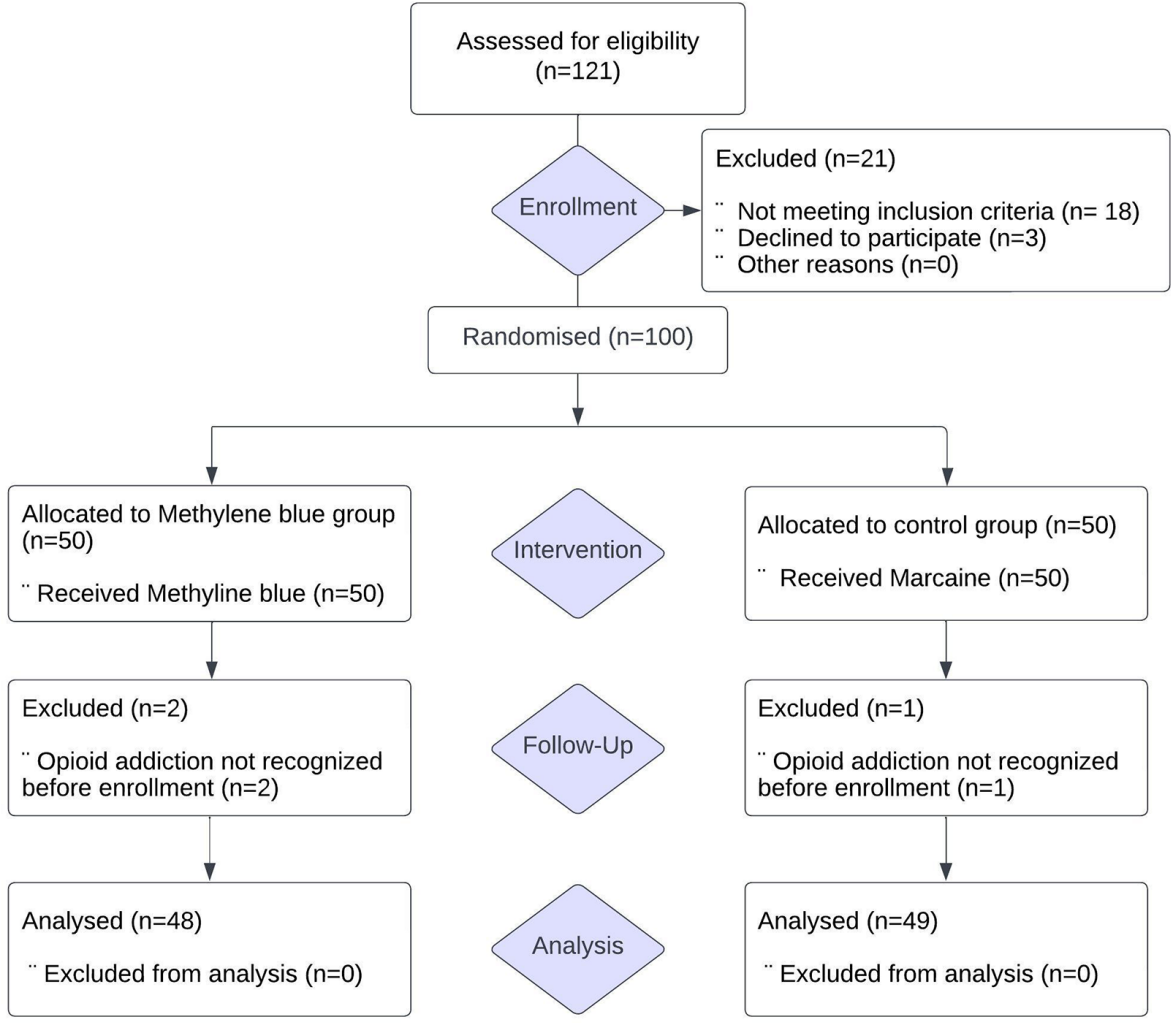
Consort flow diagram.

The study received approval from the research ethics committee of Tabriz University of Medical Sciences and was registered in the Iranian Registry of Clinical Trials under the registration number IRCT20190325043107N27 (Iranian Registry of Clinical Trials). All procedures conducted in this study adhered to the ethical standards of the institutional and/or national research committee, following the principles of the 1964 Helsinki declaration and its subsequent amendments or comparable ethical standards. Of 100 recruited for this trial, 3 were excluded because of opioid addiction not recognized before their enrolment in the trial, leaving 97 patients (48 patients in Methylene blue group and 49 patients in control group) for analysis (Figure 1).

### 2.2 Study design

In this single-center prospective double-blind randomized controlled trial conducted at Imam Reza Hospital in Tabriz, Iran, from May to September 2022, all participants underwent a standard Milligan-Morgan (19) open hemorrhoidectomy performed under a same protocol of spinal anesthesia. To ensure procedural uniformity, the same surgical team, led by a single surgeon, conducted the excision procedure for all patients. In this study, we used tailored hemorrhoidectomy with scissors and we have removed only the pathological piles (20). Randomization into two groups was achieved using a computer-generated table of random numbers (1–2), and an unbiased individual assigned patients to the intervention without knowledge of the randomization code or involvement in subsequent treatment. Both patients and investigators, including the statistician and assessor, remained blinded to allocation and randomization codes until the conclusion of the trial. Intradermal injections were administered post-surgery, immediately following the conclusion of hemorrhoidectomy, prior to patients transitioning to the recovery room. This intervention occurred during the period when patients were still under spinal anesthesia, in accordance with the study intervention protocol. Medications were intradermally injected into perianal region, specifically targeting the intradermal nerves responsible for transmitting pain sensory signals.

### 2.3 Participants

Patients aged between 18 and 75 diagnosed with stage III or IV hemorrhoids based on Goligher's classification (21), and deemed indicated for hemorrhoidectomy were included. Exclusion criteria were as follows; pregnant or breastfeeding individuals, those with adverse reactions to medications included in the protocol (adrenaline, bupivacaine), perianal infection, concurrent anorectal diseases (e.g., fissures, fistulae), a history of perianal surgery, opioid addiction, contraindications to methylene blue such as experienced anaphylaxis or other severe hypersensitivity reactions to methylene blue, relatively contraindicated in patients with glucose-6-phosphate dehydrogenase (G6PD) deficiency, individuals who are taking serotonergic psychiatric medications, renal impairment and methemoglobinemia. Informed written consent was obtained from all enrolled patients prior to their inclusion in the study.

### 2.4 Intervention

**Figure 2:**
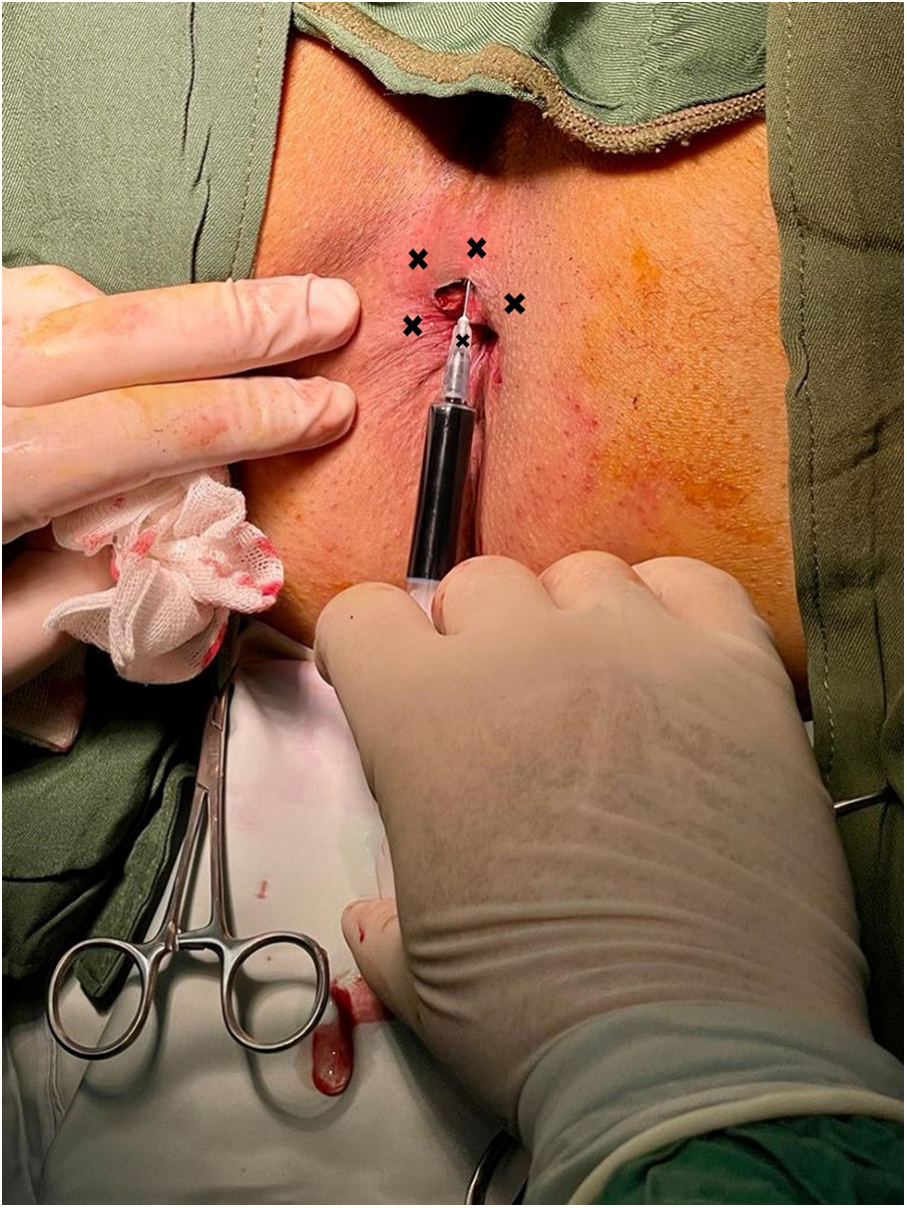
Intradermal injection of methylene blue. The procedure was executed at five distinct and evenly spaced intervals, consisting of injections of 1 ml each, positioned 2 cm from the edge of the incision, with the central point located in the incision area.

After the conclusion of the hemorrhoidectomy surgery in the operating room, an intradermal injection of 5 ml 1% methylene blue was administered to patients in the Methylene blue group (Figure 2), while the control group received an intradermal injection of 5 ml of 0.5% Marcaine in the perianal area. The intervention was performed in five separate, equal intervals, involving 1 ml injections positioned 2 cm away from the incision edge, with the central point being the incision area. The needle was inserted with an orientation of approximately 45° relative to the skin.

### 2.5 Postoperative care and follow-up

Both groups had postoperative analgesia with Ketorolac, 30 mg Endo Venous (EV) every 6 h. In the case of a patient experiencing excessive or unbearable post-operative pain, tramadol hydrochloride, 100 mg, was administered intravenously (IV) every 8 h as a rescue painkiller. All patients were discharged with prescriptions of Naproxen, 250 mg every 6 h, and acetaminophen* *+* *codeine, 300/20 mg every 4 h. Patients were meticulously instructed on postoperative wound care, emphasizing the importance of cleaning the surgical site twice daily. This involved using a mild antiseptic solution and following gentle, circular motions to maintain optimal hygiene and support the healing process. Patients were guided on the utilization of the Numeric Rating scale (NRS). While at home, individuals were instructed to document their pain levels before going to bed, noting the maximum pain experienced throughout that day. Additionally, patients kept a record of the total number of painkiller tablets consumed on each day (22, 23). To ensure greater accuracy in recording participant data for this study, daily evaluation of maximum pain scores and the use of analgesic medication were documented through telephone contact by one of the assessors. These records were collected during each visit over the preceding 14 days after the surgery. A follow-up appointment was conducted at the clinic 2 weeks after the surgery to assess the gathering of records and identify any potential complications or any notable occurrences. This assessment was done according to a checklist we provided, which will be discussed further.

### 2.6 Statistical analysis

The data analysis was conducted using R programming language version 4.2.1. Descriptive statistics were performed to describe the data based on their distribution. A sample size of 50 individuals in each study group was determined through power analysis, aiming for 90% power and a 5% significance level. Categorical variables are presented as counts and percentages, while continuous variables are expressed as mean* *±* *standard deviation (SD). Quantitative data were analyzed using Student's *t*-test for normally distributed data, and the Mann-Whitney *U*-test was employed for non-normally distributed data. A *P*-value of less than 0.05 was considered statistically significant for any observed differences.

## 3 Results

**Figure 3:**
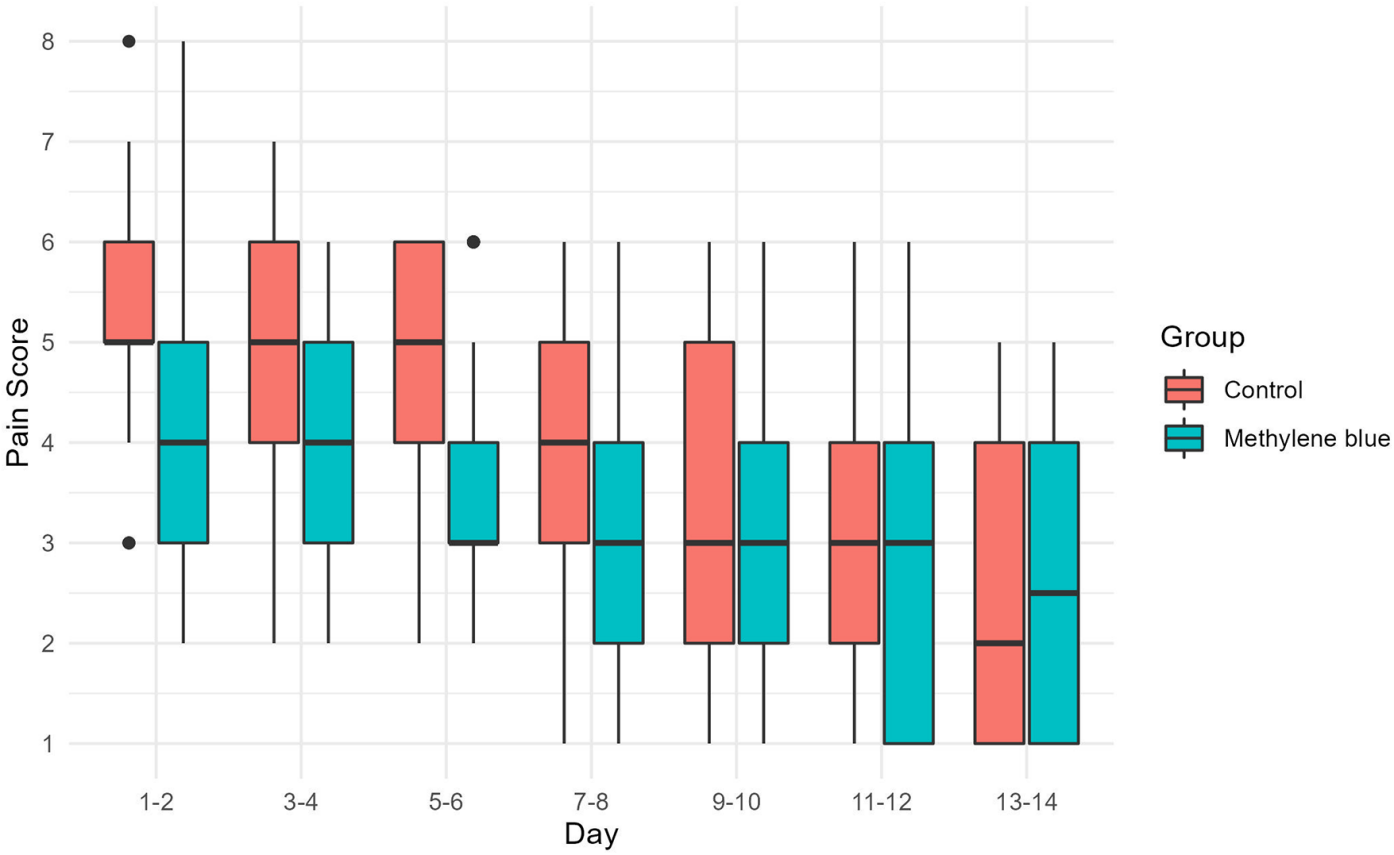
Pain score distribution during 14 days post-surgery.

**Figure 4:**
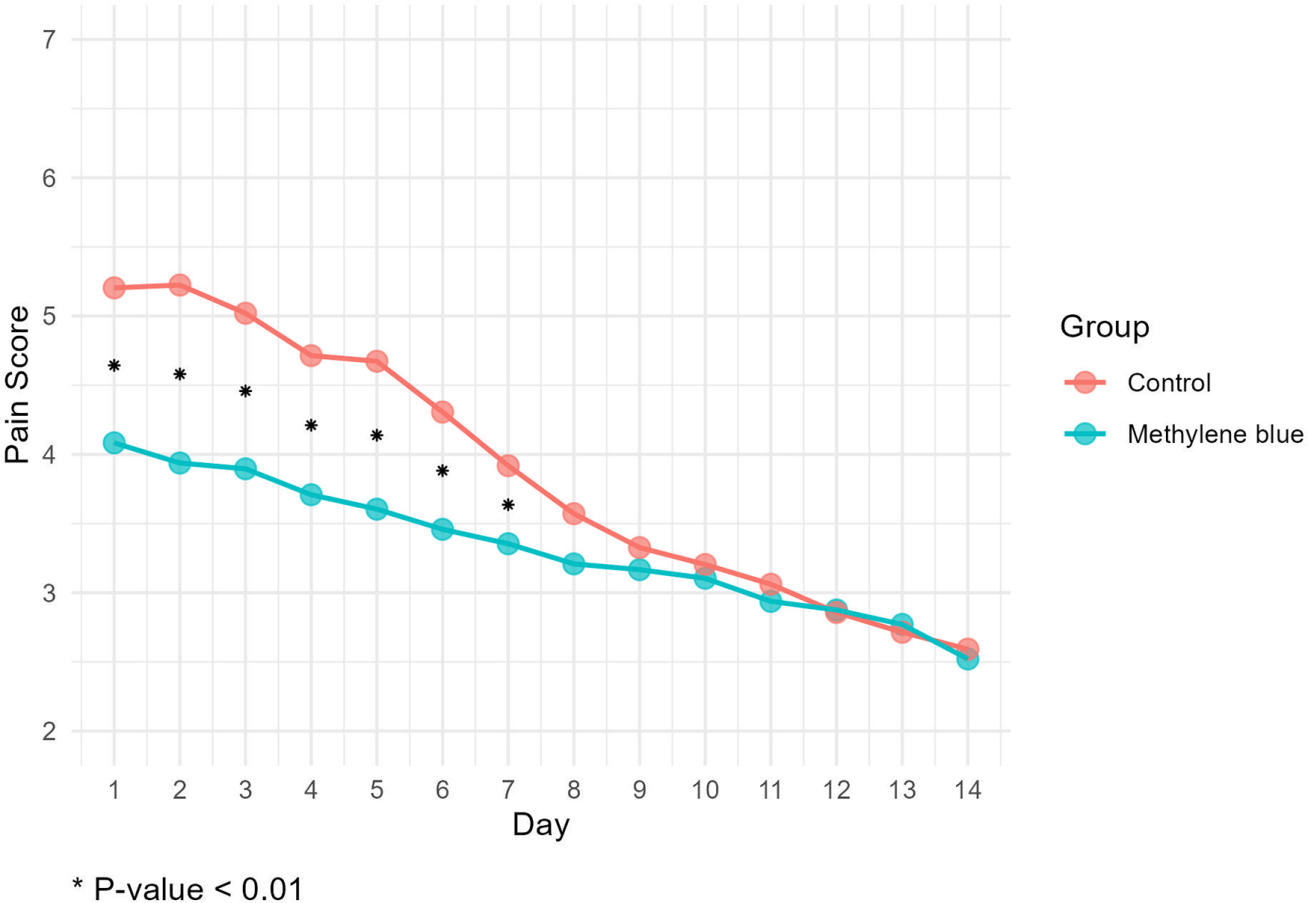
Mean maximum pain score according to numeric rating scale (11-point NRS) at first two weeks post-surgery.

We collected data from all patients diagnosed with stage III or IV hemorrhoids admitted to Imam Reza Hospital from May 2022 until we reached a total of 100 participants. During the participant selection process, 18 individuals did not meet the criteria, and an additional 3 declined to participate. By the end of August 2022, we successfully gathered a total of 100 eligible participants. There were no significant differences between two groups in terms of gender distribution (*P *=* *0.219), mean age (*P *=* *0.154), mean weight (*P *=* *0.947), mean height (*P *=* *0.559), and mean BMI (*P *=* *0.545) of patients between two group. There were also no significant differences in the number of hemorrhoids excised (*P *=* *0.616), days of hospital stay (*P *=* *0.622), and duration of surgery (*P *=* *0.328). Table 1 presents the baseline demographic and clinical data for both groups. Reported maximum pain scores are visualized (Figure 3), illustrated that maximum pain was described more than 1 score lower in at least half of the intervention group participants in compare to control group in the first 8 days post-surgery. Postoperative maximum experienced pain in the Methylene blue group was statistically significantly lower than the control group between the 1st and 7th days after surgery (*P *<* *0.01). Mean maximum pain score along Methylene blue group was lower until the 12th day. However, no significant difference in maximum pain scores was detected between the two groups from the 8th to the 14th days after surgery (*P *>* *0.05) (Figure 4 and Table 2). Notably, there were no statistically significant differences in complication rates between the two groups, as outlined in Table 3.

**Table 1:** Demographic and basal clinical features.

	Methylene blue (*n* = 48)	Control (*n* = 49)	*P*-value
Age (years)	46.02 ± 9.90	48.57 ± 7.40	0.154
Male gender	37 (75.5%)	41 (85.4%)	0.219
Height (cm)	170.29 ± 7.43	171.16 ± 7.21	0.559
Weight (Kg)	79.25 ± 7.47	79.34 ± 6.90	0.947
BMI	28.25 ± 3.95	27.79 ± 3.41	0.545
Number of hemorrhoids excised	2.33 ± 0.48	2.29 ± 0.46	0.616
Duration of surgery (min)	26.46 ± 2.90	25.86 ± 3.12	0.328
Hospital stay (days)	0.46 ± 0.50	0.41 ± 0.50	0.622

**Table 2:** Mean scores of pain intensity of post-surgery pain at different measurement times^a^.

Measurement time	Methylene blue (*n* = 48)	Control (*n* = 49)	*P*-value
Day 1	4.08 ± 1.23	5.20 ± 1.4	<0.001
Day 2	3.94 ± 1.17	5.22 ± 0.87	<0.001
Day 3	3.90 ± 1.10	5.02 ± 0.95	<0.001
Day 4	3.71 ± 1.25	4.71 ± 1.21	<0.001
Day 5	3.60 ± 1.27	4.67 ± 1.21	<0.001
Day 6	3.46 ± 1.27	4.31 ± 1.21	<0.001
Day 7	3.35 ± 1.27	3.92 ± 1.21	<0.001
Day 8	3.21 ± 1.47	3.57 ± 1.57	0.237
Day 9	3.14 ± 1.55	3.33 ± 1.57	0.636
Day 10	3.10 ± 1.60	3.20 ± 1.51	0.7189
Day 11	2.94 ± 1.62	3.06 ± 1.52	0.6403
Day 12	2.88 ± 1.50	2.86 ± 1.47	0.9794
Day 13	2.77 ± 1.43	2.71 ± 1.50	0.8017
Day 14	2.52 ± 1.43	2.59 ± 1.48	0.8113

^a^ Data is presented in the form of mean ± standard deviation.

**Table 3:** 14 days follow-up complications.

	Methylene blue (*n* = 48)	Control (*n* = 49)	*P*-value
Urinary retention	5 (10.4%)	5 (10.2%)	0.973
Pain requiring readmission	0 (0%)	1 (2%)	0.320
Excessive pain requiring unscheduled visit	1 (2.1%)	2 (4.1%)	0.570
Skin necrosis	0 (0%)	0 (0%)	NA
Anal incontinence temporarily	3 (6.3%)	1 (2%)	0.297
Wound infection	0 (0%)	0 (0%)	NA
Local skin reaction	0 (0%)	0 (0%)	NA
Pruritus	1 (2.1%)	1 (2%)	0.988
Secondary haemorrhage	0 (0%)	2 (4.1%)	0.157
Stenosis	0 (0%)	0 (0%)	NA

NA, not applicable.

## 4 Discussion

Considering that over 80% of patients experience moderate to severe postoperative pain, there is a pressing need to emphasize the significance of postoperative analgesia (24). Until now, numerous studies have been undertaken to explore effective strategies for managing pain after hemorrhoidectomy (18, 24–29). Nevertheless, the persistent issue of inadequate postoperative pain relief remains a significant concern, even with the adoption of multimodal pain management strategies (30). Different approaches and interventions for reducing pain following to excisional hemorrhoidectomy were recognized and grouped into four categories: anesthetic approaches, surgical methods, intraoperative and postoperative interventions.

In a recent systematic review and meta-analysis of seven randomized controlled trials involving 440 patients undergoing excisional hemorrhoidectomy, two anesthesia methods were compared: local anesthesia with intravenous sedation and spinal anesthesia. The findings revealed that patients receiving local anesthesia with intravenous sedation experienced significantly lower pain scores at both 6 and 24 h post-surgery. The mean differences in numerical pain rating scale scores were −2.25 (95% CI −3.26 to −1.24) and −0.87 (95% CI −1.33 to −0.40) at 6 h and 24 h, respectively (31). In relation to spinal anesthesia, it is noteworthy that adding midazolam or morphine to bupivacaine in spinal anesthesia resulted in better pain control in the first 12–24 h after hemorrhoidectomy (32, 33).

A comparison between closed (Ferguson) hemorrhoidectomy and open (Milligan-Morgan) hemorrhoidectomy regarding postoperative pain has been ongoing through randomized studies since the early 1990s (34). A recent systematic review and meta-analysis of 11 randomized controlled trials (RCTs), involving 1,326 patients (663 in closed hemorrhoidectomy and 663 in open hemorrhoidectomy), confirmed the advantage of the closed technique in reducing post-hemorrhoidectomy pain. The analysis demonstrated a modest but statistically significant reduction in pain associated with the closed technique, with a standardized mean difference of −0.36 (95% CI −0.64 to −0.07) (35).

In an examination of a hypothesis that post-hemorrhoidectomy pain may be induced by spasm in the internal anal sphincter (IAS), some studies have explored the intraoperative injection of Botulinum Toxin A into the IAS. This approach aims to induce a temporary relaxation of the IAS as an alternative to surgically dividing it, which could potentially lead to long-term issues like fecal incontinence. However, the impact of botulinum toxin injection on post-hemorrhoidectomy pain is not conclusively established, as findings from several RCTs are conflicting. Only two of these studies reported a significant reduction in post-hemorrhoidectomy pain among patients treated with botulinum toxin injection (36, 37).

In the context of minimizing opioid consumption in post-laparoscopic surgery pain management, in a study by Mulita et al. findings indicate that combinations of pethidine/paracetamol and parecoxib/paracetamol exhibit comparable analgesic effectiveness, surpassing the efficacy of paracetamol monotherapy for postoperative pain management following laparoscopic cholecystectomy. Consequently, their study substantiates the concept of a substantial opioid-sparing effect of NSAIDs in the context of postoperative pain management (38).

The analgesic activity of methylene blue involves temporarily interfering with sensory nerve conduction at nerve endings with pain and itch receptors in the epidermis and dermis. A study demonstrated that injecting 1% methylene blue 4 ml intradermally at the open hemorrhoidectomy site effectively alleviated pain during the initial 3 days after surgery, without introducing any additional complications (18). In a recent retrospective study of 180 patients with grade III or IV hemorrhoids, the effectiveness and safety of postoperative subcutaneous injection of different concentrations of methylene blue were assessed. The results indicated that both 0.1% and 0.2% perianal injections of methylene blue have equivalent analgesic effects in managing post-hemorrhoidectomy pain. Importantly, there were no significant differences in complications between the two concentrations. This study concludes that perianal subcutaneous injection of 0.1% methylene blue is not only as effective as 0.2% in treating post-hemorrhoidectomy pain but also safer (39). Accordingly, our study aimed to investigate the impact of an intradermal injection of methylene blue on postoperative pain management following hemorrhoidectomy compared to a control group receiving Marcaine. The study also explored post-surgery analgesic drug consumption and complication rates associated with each intervention.

This study shows that the NRS daily maximum pain score within 7 days in Methylene blue group were significantly lower than control group indicating that local intradermal injection of 1% methylene blue have better potential to be utilized in posthemorrhoidectomy pain management especially within the first week. We selected NRS scale because it is not only more easily applicable and understandable for patients than other pain intensity scales but also offers additional advantages. In contrast to alternative scales like the Visual Analogue Scale (VAS), the NRS utilizes a broader range of ratings (0–10), making it a more sensitive tool for accurately assessing changes in pain intensity (40). According to results of the present study, there was no significant difference in complications among groups, including urinary retention, skin necrosis, wound infection, secondary haemorrhage, and pruritus. This indicates that 1% methylene blue intradermal injection after hemorrhoidectomy has little effect on anal function, and it is temporary and reversible. Additionally, drug reverse reaction or short time systemic or local side effects did not seen in neither of groups, indicating that methylene blue has unremarkable side effects and is not only effective but also relatively safe to be used in wider aspects.

However, there are still some limitations to be considered in this study. first of all, there was small samples size and short follow-up period of only 2 weeks. We should continue to expand the sample size and conduct long-term follow-up analysis of patients. Much larger sample size and follow- up period are needed to achieve more accurate estimations in effectiveness and probable side effects or complications.

## 5 Conclusion

Current randomized controlled trial shed light on potential analgesic effect of 1% methylene blue. Methylene blue has a comparable efficacy in managing posthemorrhoidectomy pain with unremarkable side effects and complications to be utilized in future more extended aspects.

## Data Availability

The raw data supporting the conclusions of this article will be made available by the authors, without undue reservation.
